# The role of spatial perception in auditory looming bias: neurobehavioral evidence from impossible ears

**DOI:** 10.3389/fnins.2025.1645936

**Published:** 2025-08-25

**Authors:** Tobias Greif, Roberto Barumerli, Karolina Ignatiadis, Brigitta Tóth, Robert Baumgartner

**Affiliations:** ^1^Acoustics Research Institute, Austrian Academy of Sciences, Vienna, Austria; ^2^Department of Neurosciences, Biomedicine and Movement, University of Verona, Verona, Italy; ^3^Institute of Cognitive Neuroscience and Psychology, HUN-REN Research Centre for Natural Sciences, Budapest, Hungary

**Keywords:** EEG, spatial hearing, auditory distance, externalization, auditory looming bias, sound localization, perceptual decision-making

## Abstract

**Introduction:**

Spatial hearing enables both voluntary localization of sound sources and automatic monitoring of the surroundings. The auditory looming bias (ALB), characterized by the prioritized processing of approaching (looming) sounds over receding ones, is thought to serve as an early hazard detection mechanism. The bias could theoretically reflect an adaptation to the low-level acoustic properties of approaching sounds, or alternatively necessitate the sound to be localizable in space.

**Methods:**

To investigate whether ALB reflects spatial perceptual decisions or mere acoustic changes, we simulated ears that disrupted spectrospatial associations on the perceptual level while maintaining the original spectrospatial entropy on the acoustic level. We then assessed sound localization, ALB and distance ratings.

**Results:**

Compared to native ears, these novel ears impaired sound localization in both the direction and ego-centric distance dimensions. ALB manifestation also differed significantly between native and novel ears, as evidenced by behavioral discrimination performance and early cortical activity (N1 latency). Notably, the N1 electroencephalographic response closely resembled distance ratings, suggesting a strong link between spatial perception and ALB-related neural processing. Integrating this neural marker into a hierarchical perceptual decision-making model improved explanatory power, underscoring its behavioral relevance.

**Discussion:**

These findings suggest a strong link between the localizability of sounds and their ability to elicit ALB.

## 1 Introduction

Spatial hearing is a natural and integral part of the human sensory experience. Being able to make inferences about the location of a sound source is an important skill to navigate throughout the world (Middlebrooks, 2015; Majdak et al., 2020), as is the automatic and continuous monitoring of our surrounding auditory space in order to detect potential incoming dangers. One key example for this threat detection functionality is the auditory looming bias (ALB), which describes a perceptual preference for looming, i.e., approaching, sounds, compared to receding ones, presumably caused by evolutionary adaptation (Neuhoff, 2001; Ignatiadis et al., 2024a; Neuhoff, 2018). The auditory system, in a sense, “warns” the organism that something is approaching and enables it to react quickly and appropriately by, for instance, moving out of the way. This adaptive nature is also highlighted by traditional ALB experiments showing that looming, compared to receding stimuli, exhibit significantly faster reaction times as well as better discrimination accuracy (Bach et al., 2009; Baumgartner et al., 2017; Bidelman and Myers, 2020; Ignatiadis et al., 2024a; McCarthy and Olsen, 2017; Neuhoff, 2001, 1998; Neuhoff et al., 2009; Seifritz et al., 2002). It has also been suggested that ALB elicitation is contingent on the perception of a continuous auditory object moving in distance (Baumgartner et al., 2017), but the exact details behind it are yet poorly understood. In particular, current evidence is inconclusive as to whether low-level acoustic features, typically emitted by approaching sound sources, trigger the bias or whether higher-order perceptual processes drive it instead.

Most ALB studies (Bach et al., 2009; Bidelman and Myers, 2020; McCarthy and Olsen, 2017; Neuhoff, 2001, 1998; Neuhoff et al., 2009; Seifritz et al., 2002) approximated the sensation of looming and receding sound sources solely by manipulating overall sound intensity, i.e., increasing intensity to signal looming; decreasing intensity to signal receding. A neurobehavioral study expanded on this by demonstrating that the ALB could also be elicited via spectral changes while keeping broadband intensity constant (Baumgartner et al., 2017). By transitioning between two sounds, one filtered with the listener-specific (native) head-related-transfer-function (HRTF) and the other with a spectrally flat HRTF, they were able to create looming and receding sensations and elicit the ALB. HRTFs capture spectral cues at high frequencies that strongly depend on sound-source location and are mainly induced by the complex morphology of the listener's ears (Blauert, 1996). A loss of spectral features, i.e., a flat HRTF, impoverishes cues that are central for perceptual externalization, the sensation that a sound is originating from a certain ego-centric distance in “the outside world,” as opposed to from “within-the-head”; an essential skill for accurate environmental awareness and immersive auditory perception, particularly in complex listening environments. A degradation of externalization is often observed when listening through headphones (Hartmann and Wittenberg, 1995; Jeffress and Taylor, 1961) or hearing aids (Best et al., 2020; Best and Roverud, 2024). Transitioning from a native, successfully externalized sound, into a flat, moreso internalized sound, could thus elicit the perception of an approaching, looming, object- or vice versa a receding object.

Subsequent work has since replicated ALB elicitation via spectral shaping and demonstrated that neural markers of ALB could be elicited even in a passive listening mode with diverted attention (Ignatiadis et al., 2024a). Interestingly, the observed differences between looming and receding stimuli are represented neurally rather early, already 80 ms and extending up 250 ms after motion onset. This is mostly in line with latency differences found between the processing of natural and artificial spatial cues in auditory motion perception (Getzmann and Lewald, 2010) but rather early compared to other aspects of spatial hearing relying on spectral cues. Changes in elevation, for instance, are only neurally distinct 200-400 ms after elevation-change onset (Fujiki et al., 2002; Bialas et al., 2023). In human newborns ALB markers were only found using intensity cues, not spectral cues (Ignatiadis et al., 2024a), indicating that ALB elicitation based on spectral cues seemingly depends on spatial associative learning over lifetime, suggesting a developmental reliance on more salient, low-level acoustic features prior to the maturation of spectro-spatial integration. Over the course of development, the auditory system receives input from diverse spatial locations and gradually forms associations between spectral characteristics and spatial positions (Hofman et al., 1998; Wanrooij and Opstal, 2005), likely forming an internal spectro-spatial map that guides spatial perception. However, neonates exhibit a range of unique EEG characteristics that make it substantially different from an adult EEG, such as diffuse slowing, discontinuity, asynchrony and minimal reactivity (Louis et al., 2016), which complicates the interpretation of ERP markers like N1 or MMN that are typically used to assess spatial auditory processing.

Previous work has also argued that not only spatial perceptual associations but also the identifiability of the sound contribute to ALB elicitation. When presenting intensity ramps diotically over headphones, natural tonal/harmonic sounds have been reported to elicit ALB more effectively than noise, with some authors arguing that this might be due to the more realistic nature of tonal stimuli, perhaps resembling the sounds of a predator more closely and, generally, being more easily identifiable (Neuhoff, 1998, 2001; Ponsot et al., 2015). An alternative interpretation of these results, however, would be that the diotic presentation negatively impacted bias elicitation with noise. Broadband sounds, such as noise, are expected to provide spectral localization cues to listeners. When these cues are absent due to diotic presentation, the spatial percept is expected to degrade (Baumgartner and Majdak, 2021), hindering ALB elicitation since the sound is less externalized to begin with and little to any motion would be perceived. Tonal sounds, lacking sufficient bandwidth, cannot convey spectral localization cues in the first place and therefore do not lead to such conflicts that might interfere with bias elicitation. Therefore, the reported stimulus dependencies favoring tonal/harmonic diotically presented sounds may instead reflect the necessity of spatial perceptual associations to elicit ALB. In line with this, other studies have since reported stimulus dependencies in the opposite direction, favoring noise over tonal/harmonic stimuli, when investigating ALB elicitation using different tasks (McCarthy and Olsen, 2017) or species (Deneux et al., 2016), suggesting that perhaps the additional spectral localization cues aided in ALB elicitation. However, these inconsistencies in the literature make it difficult to decisively conclude whether the (spectral) ALB is truly a spatial phenomenon and depends on learned associations between spatial positions and spectral cues, driven by a change in externalization, or whether spectral properties/changes themselves are sufficient to facilitate the bias. The previously reported early processing latencies suggests that the ALB might constitute a pre-attentive mechanism that possibly reflects rapid sensory prioritization. However, the discrepancies in terms of stimulus dependencies, as well as the absence of a spectral ALB in newborns, call this into question.

**Figure 1:**
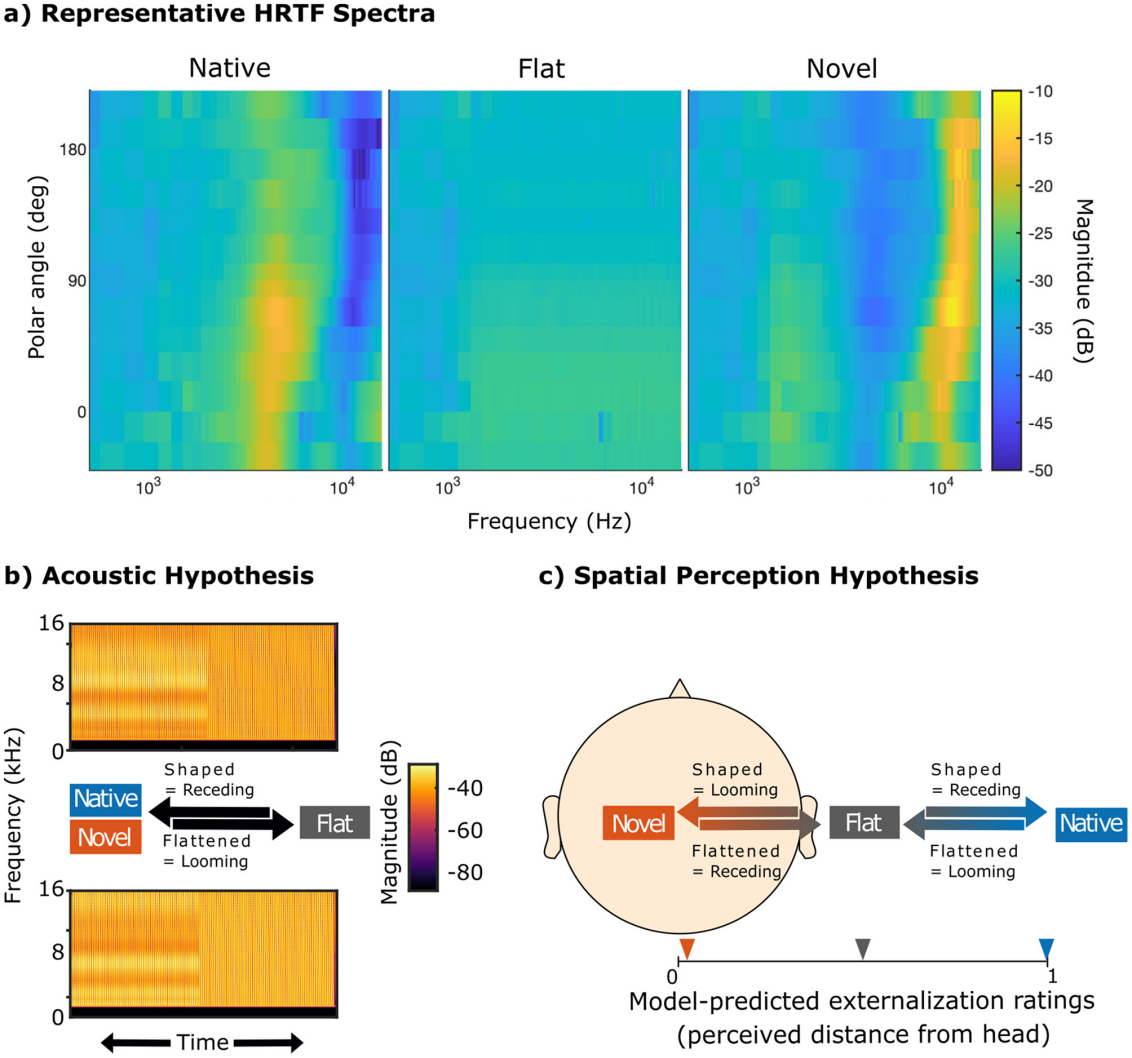
Illustration of the spectral-shape cue manipulations and alternative hypotheses regarding ALB elicitation. “Native,” “novel,” and “flat” refer to participant's HRTFs; “shaped” and “flattened” refer to the stimulus manipulation based on the transition between HRTFs; “looming” and “receding” refer to the expected percept under the respective hypothesis. **(a)** Spectral-shape cues along the median plane for an example participant. **(b)** Acoustic Hypothesis: flattened stimuli (decreasing magnitude of spectral contrast factor *C*), regardless of spectral cue type, elicit stronger responses compared to shaped stimuli (increasing magnitude of spectral contrast factor *C*). If this hypothesis holds, then previous studies may have mistakenly interpreted neural biases as ALB. **(c)** Spatial Perception Hypothesis: stimuli that exhibit a reduction in perceived externalization are perceived as looming and elicit ALB as opposed to stimuli that exhibit an increase in perceived externalization. If novel HRTFs are less externalized than flat HRTFs, as suggested by model predictions based on our participant's HRTFs (Baumgartner and Majdak, 2021), novel shaped stimuli would thus be perceived as looming instead of receding.

The present study aimed to investigate how spectral cues of novel, physically impossible ears, intended to disrupt established spectrospatial associations while providing a comparable amount of spectral information (Figure 1a), affect directional localization, externalization, and ALB elicitation. We expected novel HRTFs to acutely degrade localization performance (Middlebrooks, 1999) and externalization perception (Hartmann and Wittenberg, 1995). We hypothesized ALB elicitation to be in-line with changes in externalization rather than the amount of spectral shaping (Baumgartner et al., 2017). If the ALB is driven by the amount of spectral shaping rather than spatial associations, any transition into a flat HRTF should elicit the ALB. That is, both novel into flat as well as native into flat stimuli should elicit the ALB (Acoustic Hypothesis, Figure 1b). If, on the other hand, the bias relies on spatial associations, it should be consistent with the change in externalization perception. As native spectral cues are thought to form the reference and increasing deviations from this reference are expected to degrade externalization, the externalization model proposed by Baumgartner and Majdak (2021) predicts that flat HRTFs are less externalized than native HRTFs, but also that novel HRTFs are even less externalized than flat HRTFs (Spatial Perception Hypothesis, Figure 1c). If this prediction holds, stimulus transitions from novel to flat would instead be perceived as receding, and thus elicit a bias in the opposite direction than the native case. Third, we hypothesized early neurophysiological markers of the ALB, such as ERP components associated with motion onset, to be behaviorally relevant, suggesting a shared underlying mechanism. The relationship between behavioral and neural ALB measures was assessed via joint hierarchical modeling (Turner et al., 2013; Brown and Heathcote, 2008).

## 2 Methods

Normal-hearing young adults performed three spatial hearing tasks. First, behavioral localization experiments were conducted in virtual reality (Majdak et al., 2010), then ALB was tested using a neurobehavioral (EEG) paradigm (Baumgartner et al., 2017), and finally perceived sound externalization was rated on a continuous scale (Boyd et al., 2012; Leclère et al., 2019). We contrasted neurobehavioral responses to native spectral cues against responses to novel spectral cues that provided a similar amount of spectral change but were designed to disrupt established spectrospatial associations.

### 2.1 Participants

We recruited 15 young adults (10 male; mean age: 25.6 ± 2.19) from the institutes' internal participant database. The sample size was chosen identical to a previous study (Baumgartner et al., 2017). One participant was excluded from the ALB and localization analyses due to a trigger box malfunction during the EEG recording and missing data. Externalization data were only collected from the last 11 participants. All participants had normal or corrected-to-normal vision as well as normal hearing, meaning that audiometric hearing thresholds did not differ more than 20 dB from their age-specific norm (Rodríguez Valiente et al., 2014) in the high-frequency frequency range of 1 to 16 kHz. All participants were monetarily compensated for their participation. The study applied the standard methodology of the Acoustics Research Institute, which the Ethics Board of the Austrian Academy of Sciences has approved.

### 2.2 Paradigms

The order of tasks was fixed across participants. Everyone began with directional localization followed by the looming bias task and, finally, the externalization paradigm. This order was chosen deliberately because naive participants experience high difficulties when performing a spatial task in anechoic conditions. Since the localization paradigm was the most interactive, it served as a good starting point for the testing session. Externalization was only evaluated after the looming bias paradigm as we aimed to avoid priming effects that would have come about by participants actively evaluating distance perception with novel spectral cues prior to the looming bias paradigm. All participants, but not the experimenter, were blinded regarding which experimental conditions were tested at what point within each paradigm.

#### 2.2.1 Directional localization

To evaluate localization abilities for our different stimulus manipulations, we adapted an established VR-localization paradigm (Majdak et al., 2010). The experiment itself was conducted in a three-dimensional virtual environment (Oculus Rift CV1, Oculus VR). Inside the environment, a gray empty space was shown, with a red sphere indicating a central reference point in front of the participant. Around the median and coronal plane, 29 smaller blue spheres were displayed in order to allow for easier navigation in the otherwise empty space. Participants were allowed to complete the experiment either seated, or standing up. However, their movement was limited such that they could only rotate but not translate through the real physical space.

Participants initiated the start of every trial via a button press on a hand-held controller. Their head had to be oriented toward the front and kept still during stimulus presentation, as to not introduce conflicting proprioception. Once a stimulus had been presented from a randomly drawn position in space, participants used the hand-pointing device (visible to them) to indicate the direction the sound came from. Participants first completed six training blocks with native cues only (50 trials each), to become familiar with the VR setup and task procedures. Training blocks also incorporated visual and auditory feedback (Majdak et al., 2010), as well as sensorimotor feedback. Here, after giving their initial response, a red cube appeared at the actual sound-source position (visual feedback). Participants were then required to acknowledge this “true” position by selecting the cube with the hand pointer, before they were asked to fixate back on the initial mid-front position. The sound was then repeated (auditory feedback) and subsequently played continuously, allowing participants to localize it while turning their head in any direction until they confirmed its position by selecting the cube once again (sensorimotor feedback). After familiarization was completed, participants continued with the testing procedure. Here, stimuli were presented in blocks of 100 trials each, with a fixed order of native, flat and then novel. This was repeated thrice, resulting in 300 trials per condition and 900 trials in total. No feedback was provided during testing blocks and participants were free to take breaks between blocks at any point but were forced to do so after three experimental blocks to limit fatigue. Depending on the speed and break timing of individual participants, the experiment took around 3 h to complete.

#### 2.2.2 Looming bias

Participants performed a two-alternative forced-choice motion discrimination task while EEG was recorded. They were seated in a dimly lit room in front of a black computer screen with a central white fixation dot and were presented the above-mentioned spectrally shaped or flattened sounds. After each stimulus played in its entirety, participants had to judge via a keyboard button press (right index finger), whether they perceived the sound as looming or receding. Each participant completed eight experimental blocks with 100 trials each. Block order was fixed and participants alternated between native-only and novel-only blocks, starting with native-only because participants expressed difficulties with judging motion direction when starting with novel-only blocks and also when native and novel trials were interleaved during the pilot phase of the experiment. This was probably caused by the transitions between spectral cues being quite subtle and thus making the task rather difficult, especially when interleaving trials. Within each block, 50 shaped and 50 flattened stimuli were presented in a randomized fashion. In total, the experiment took around 90 min and participants received no feedback on their performance. We opted for a forced-choice task without a non-response option in order to facilitate the analysis based on linear ballistic accumulator modeling (Section 2.4.4). Although this left the possibility of trials without distance motion percepts uncontrolled within the looming bias task, we subsequently collected perceptual externalization ratings for the start and end phase of each stimulus.

#### 2.2.3 Externalization

To assess the perceived externalization of sounds filtered with different spectral contrasts, participants were seated in front of the same experimental screen. They judged every sound's perceived degree of externalization on a continuous visual sliding scale. The scale was anchored by the image of a loudspeaker on the left side and the representation of a head on the right side. The range between the ear and midpoint of the head was represented by 15% of the scale. Each combination of position and spectral contrast was judged three times, resulting in 36 ratings given. Participants were presented with 12 pages on the experimental screen containing three trials each. On each page, the three spectral contrasts were presented together, from one of the four possible positions. Participants could listen to all three sounds on one page as many times as they wanted and could adjust each scale as needed. Once they were satisfied with their judgments, they could move on to the next page, where the next position was presented. The order of positions and the order of spectral contrasts on each page was randomized and participants were blind to the experimental conditions. Overall, the externalization task took between 20 and 30 min to complete.

### 2.3 Stimuli

We first recorded the acoustic transmission properties for each participant in terms of listener-specific HRTFs that take into account the complex listener-specific pinna, head and torso geometry [apparatus and procedure described in detail in Ignatiadis et al. (2024a)]. Thus, we captured the native spectrospatial properties of sounds arriving at the ear canal, which allowed us to subsequently manipulate and create sounds with distinct spectral shapes to our needs. The measured HRTF magnitude spectrum (spectral contrast factor *C* = 1; at 1.2 m distance from the listener) was spectrally smoothened (gammatone filter with bandwidth factor of 1) (Hassager et al., 2016) to obtain native spectral cues and then either mathematically inverted (*C* = −1) to obtain novel spectral cues or deprived of spectral spatial information (*C* = 0) to obtain flat spectra by applying a previously proposed manipulation method (Baumgartner et al., 2017):


(1) $$\begin{array}{l} M_{c}\left(f\right)=CM_{1}\left(f\right)+\left(1-C\right)\frac{1}{N_{f}}\underset{k\in f}{\sum }w^{\prime }\left(k\right)M_{1}\left(k\right), \end{array}$$

where *M*_1_(*f*) denotes the magnitude spectrum of the native HRTFs, *N*_*f*_ denotes the frequency bins within the manipulated frequency range of 1–16 kHz, and *w*′ denotes a frequency weighting function that approximates auditory frequency resolution by the across-frequency derivative of equivalent rectangular bandwidths (Glasberg and Moore, 1990). Representative HRTF spectra are shown in Figure 1a.

For the directional localization experiment, 500 ms Gaussian white noise bursts with 10 ms on- and offset ramps were manipulated using the spectral contrast procedure described above. This resulted in sets of stimuli that contain either the native spectral information, novel spectral information, or are devoid of any spectral information (i.e., flat).

**Figure 2:**
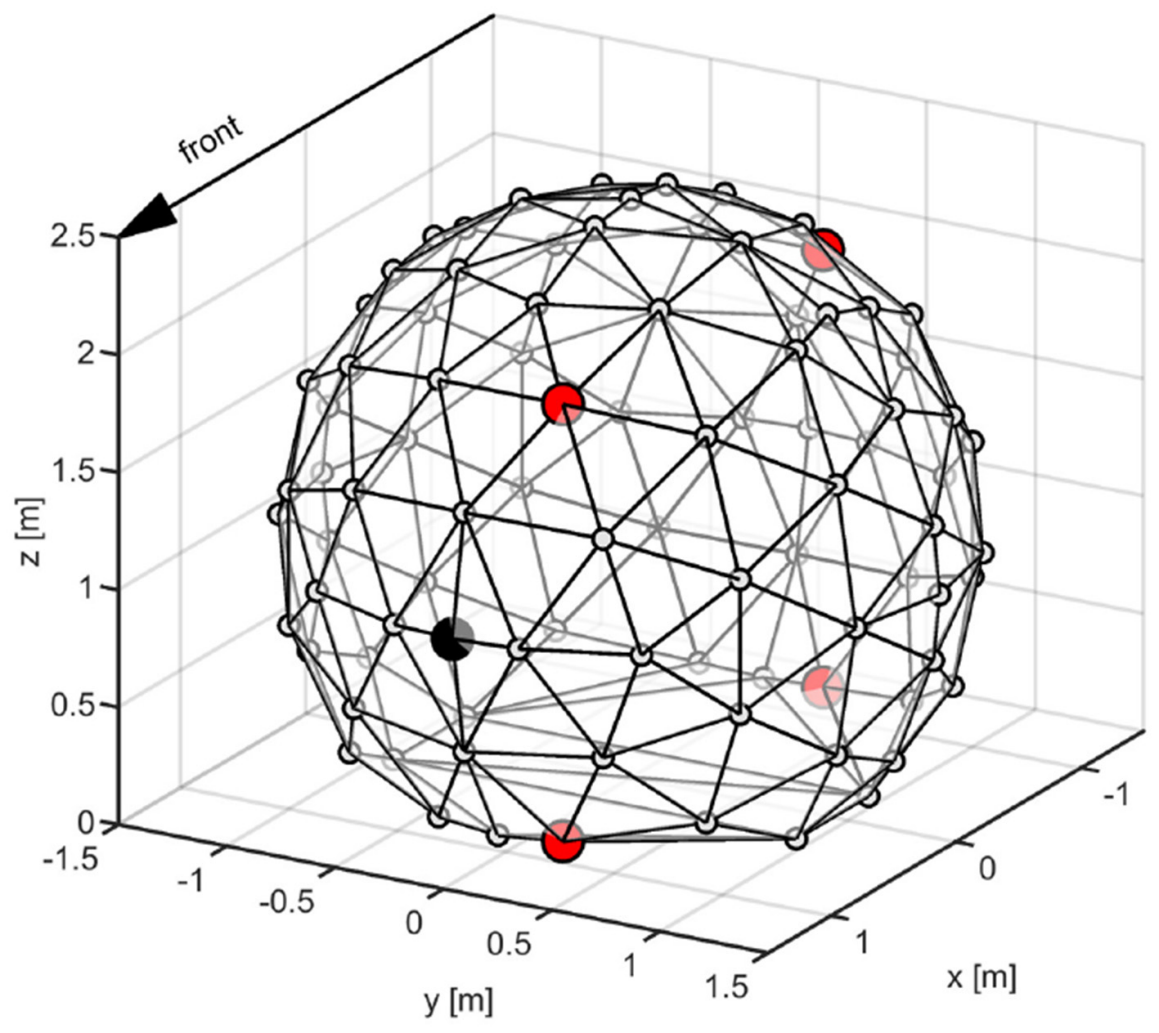
Illustration of the loudspeaker array system with 91 source positions. Participants are facing the speaker highlighted in black during the localization paradigm. Positions used for generating the ALB and externalization stimuli are highlighted in red.

For the externalization ratings, harmonic tone complexes (12 different fundamental frequencies evenly spaced between 100 and 140 Hz, bandwidth: 1–16 kHz, phase curvature: 0.5) (Schroeder, 1970) were corrected for constant loudness by iterative model-based adjustments (Moore et al., 2016) and subsequently filtered with either native, novel or flat HRTFs from four possible incidence angles, i.e. positions: Front-Up (FU) = 45°, Front-Down (FD) = 135°, Back-Up (BU) = 225° and Back-Down (BD) = 270° (Figure 2). We varied the fundamental frequency and the position of our stimuli, in order to avoid making the task too monotonous as well as enforcing listeners to base their judgements on spatial, rather than specific timbre, cues.

Looming bias was assessed using the same tonal stimuli as for the externalization ratings but combined in pairs. By transitioning between two stimuli with different spectral manipulations, we created flattened and shaped stimuli. The transition was initiated 600 ms after the onset of the first sound, with a jitter of ± 50 ms, and was implemented as a short 10 ms crossfade with raised cosine faders to ensure constant intensity for the highly correlated tonal stimuli. The transition was kept short to obtain a temporally clearly defined motion event for the ERP analysis (Baumgartner et al., 2017; Ignatiadis et al., 2024a).

All stimuli across all paradigms were presented binaurally using tubephones (ER-2, Etymotic Research) at an approximate sound pressure level of 70 dB at the ipsilateral ear.

### 2.4 Data analysis

#### 2.4.1 Directional localization

To assess localization performance of our participants, we separately analyzed the following error measures: lateral error, local polar error and quadrant error rate (global polar error) (Middlebrooks, 1999). Lateral and local polar errors were defined as the difference in angular coordinates between target and response, along both the lateral dimension ranging from left (–90°) to right (90°) and the polar dimension ranging from below (–90°) via front (0°) and above (90°) to back (180°). The local polar error excluded all polar errors exceeding 90° in absolute terms. The quadrant error rate was defined as the percentage of trials where the polar error exceeded 90°.

In order to reveal the effect of our stimulus manipulations on participants' error rates, we conducted repeated measure ANOVAs for each error measure separately. Due to the long testing sessions we additionally included an “order” factor in these analyses to check whether performance differed between the first and second half of responses for each cue type. Partial eta squared was calculated as a measure of effect size. If a significant difference between at least two groups was found, we conducted Tukey-HSD *post-hoc* tests to reveal which groups differed from each other. Sphericity assumptions were tested using Mauchly's test for sphericity, implemented in MATLAB. In case of violations, a Greenhouse–Geisser correction was applied.

#### 2.4.2 Externalization

Externalization rating data were collected on a continuous scale ranging from 0 to 1, representing a percept localized at the center of the listener's head to a fully externalized sound image at the distance of the loudspeakers used for the HRTF measurement, respectively. The data were averaged over fundamental frequencies to achieve normality for beta regression modeling. Using the R (Version 4.4.1) packages data.table (Barrett et al., 2024), emmeans (Lenth, 2024), and ggplot (Wickham, 2016), we tested five different model alternatives taking into account the influence of cue-type (native, novel, flat) and position (FU, FD, BU, BD) on externalization ratings: (1) Intercept-only; (2) main effect of cue-only; (3) main effect of position-only; (4) main effects of both cue and position, and (5) main effects of cue and position and their interaction. All model alternatives were fitted using the maximum likelihood approach and a logit-link implemented in the R package betareg (Grün et al., 2012). Model comparison was based on Bayesian information criterion (BIC). To determine the significance of main effects, a Bayes Factor (BF) approximation based on the difference in BIC between an intercept-only model and a main effect model, and the associated posterior probabilities, was computed to express evidence in favor of, or evidence against, a model including a main effect (Wagenmakers, 2007). *Post-hoc* pairwise comparisons were conducted using estimated marginal means and the *p*-values were adjusted using Tukey's multiplicity adjustment.

#### 2.4.3 EEG

EEG data were recorded using a high-density 128-channel EEG cap (actiCAP with actiCHamp; Brain Products GmbH, Gilching, Germany) and the Brain-Vision Recorder software. Pre-processing was conducted in EEGLAB (Delorme and Makeig, 2004). As a first step, bad channels (i.e., channels with high impedance (≥50*kΩ*) and visible noise during the recording) that were marked as such during data collection were removed. Subsequently, the data was bandpass-filtered (0.5–30 Hz) using an FIR filter with Kaiser window (β = 7.2, *n* = 462). Since our event of interest is the change/transition between the sounds with different spectral contrasts, the data was epoched around this changepoint (600 ± 50 ms after stimulus onset) into segments of [–100 1,500] and subsequently resampled to 100 Hz. We then performed an Independent Component Analysis (ICA) and removed components indicating eye movement-related artifacts or power-line-noise using EEGLAB's ICLabel plugin. Components that were categorized as eye movements or power-line-noise with a high probability (≥80%) were manually checked and removed. Manual visual inspection also took place on a trial-by-trial basis, removing trials that contained apparent noise artifacts such as malfunctioning channels that were not noted previously. No automated trial and/or channel rejection took place. A maximum of ten malfunctioning EEG channels and 5% excluded trials were accepted per participant. On average, 2.14 (SD: 1.46) channels and 7.43 (SD: 6.55) trials were rejected per participant. After data cleaning was completed, the missing channels were interpolated using EEGLAB's default spherical interpolation function. The data were then re-referenced to the average. Next, we equalized the number of trials to retain the same amount across all conditions within participants. This was done by finding the minimum number of trials present within all conditions, and then rejecting trials from the other conditions, equally spaced across the session. This resulted in, on average, 0.7 (SD: 1.12) trials being removed per condition per participant and ensured an unbiased, blind trial equalization procedure.

For statistical inference, we used Fieldtrip (Oostenveld et al., 2010). Baseline correction was performed on each individual trial, using the 100 ms before the event of interest. The data was organized into group-level structures and average ERPs for our four conditions were computed with ft_timelockanalysis. To address our research question, we first compared the evoked potentials for flattened and shaped stimuli for the native and novel conditions separately. Next, we compared the evoked flattened-shaped contrast for native cues with the one for novel cues, by subtracting the shaped condition from the flattened condition, separately for native and novel, and then comparing those differences. To address whether there were any significant spatiotemporal differences between our conditions, we used non-parametric cluster-based permutation testing (Maris and Oostenveld, 2007). Neighbors were defined using Fieldtrip's “distance” method. The test-statistic of choice was a two-tailed dependent-samples t-test, using the Monte Carlo re-sampling method (10,000 permutations; alpha: 0.05). Our analysis time-window was restricted to 0–300 ms. To account for the two-tailed test, the resulting probabilities were corrected using Fieldtrip's correcttail function set to prop. Aggregated cluster scores were calculated by averaging over the whole cluster epoch and all channels that contributed to the cluster at any point. Finally, we conducted a two-way repeated measures ANOVA to investigate the effects of spectral cue and position on the evoked native-novel difference within the obtained cluster. Sphericity assumptions were again tested using Mauchly's test for sphericity. In case of violations, a Greenhouse–Geisser correction would have been applied.

#### 2.4.4 Neurobehavioral model

We evaluated the ALB magnitude in the behavioral data of the looming bias paradigm by combining response choices and reaction times within a linear ballistic accumulator (LBA) model (Brown and Heathcote, 2008), implemented in R (Version 4.4.1) using the packages EMC2 (Stevenson et al., 2024), data.table (Barrett et al., 2024), msm (Jackson, 2011), coda (Plummer et al., 2006), and ggplot2 (Wickham, 2016). The LBA model is designed for multi-alternative forced choice tasks, where each possible response option competes with the others by accumulating evidence at a specific rate, known as the drift rate *v*_*i*_. Each accumulator starts from a random starting point drawn from a uniform distribution within the range [0, *A*]. A response is generated when the first accumulator crosses a decision threshold *B*. To account for residual variance in total response times, such as motor or sensory latencies, the model also includes a non-decision time parameter *t*0. Performance in forced choice tasks has been demonstrated to reflect the combined effects of task difficulty and response caution; hence, the LBA model jointly accounts for both speed and accuracy (or the likelihood of a certain response) and can thus control for the speed-accuracy trade-off present in forced choice tasks (Brown and Heathcote, 2008). In contrast to process-oriented models such as the LBA, analyzing accuracy and reaction times separately might lead to misleading results when interpreting changes in distributional parameters (e.g., mean location) as variations of the underlying cognitive processes (Matzke and Wagenmakers, 2009).

For parameter estimation, we employed Bayesian statistics with a Markov Chain Monte Carlo (MCMC) approach, enabling hierarchical parameter estimation. This method provided robust estimates and facilitated the analysis of group-level parameter variations (Gunawan et al., 2020). In particular, we relied on the library EMC2 (Stevenson et al., 2024), which implements the particle Metropolis within Gibbs (PMwG) approach for MCMC. This approach achieves faster convergence, requiring fewer samples compared to earlier methods (Gunawan et al., 2020). Convergence was ensured through multiple chains (independent sequences of samples), allowing for automatic convergence assessment based on the Gelman-Rubin diagnostic (Brooks and Gelman, 1998). We used default EMC2 prior values and, after parameter estimation, inspected the contraction statistics (i.e., one minus the ratio of posterior sample variance to prior sample variance) to verify minimal prior influence.

We employed linear modeling features to examine how experimental factors influenced model parameters across participants. Our experimental design incorporated spectral cue type (native or novel), motion direction (flattened or shaped), stimulus-response match (flattened → looming or shaped → looming), and position. For position, we partitioned the levels into two factors: vertical (up or down) and horizontal (front or back). This allowed us to test multiple configurations of experimental factors and their relationships with model parameters. Quality of fit was compared across models using the Bayesian Predictive Information Criterion (BPIC) metric (Ando, 2011), which aims to balance model fit and parsimony. By penalizing models with excessive free parameters, BPIC helps mitigate overfitting.

In addition to using only behavioral data, we employed a joint modeling approach to test whether integrating EEG data in the model's parameter estimation improves its quality of fit (Turner et al., 2013). For this configuration, we extracted the mean amplitudes of the EEG signal within a significant spatiotemporal cluster of difference (see Section 3.3) on a trial-by-trial basis and included them as an additional regressor to predict the drift rates. The method follows a simple directed approach that includes the cluster amplitude as a linear predictor of cognitive parameters. If the inclusion of this EEG-based regressor improves the model's predictive power, it supports the assumption that the extracted EEG activity is relevant to the behavioral outcome. Thereby, the joint approach enables a more comprehensive understanding of how perceptual decisions originate from neural activity (Turner et al., 2013).

To assess differences in drift rates between factors, we sampled drift rates from the group-level posterior distributions and computed the mean difference between conditions, denoted Δ. This quantity served as a summary statistic to capture the direction and magnitude of drift rate changes. We then estimated its 95% credible interval and the probability of Δ being positive or negative using a one-tailed credible interval approach (McElreath, 2018).

## 3 Results

### 3.1 Directional localization

**Figure 3:**
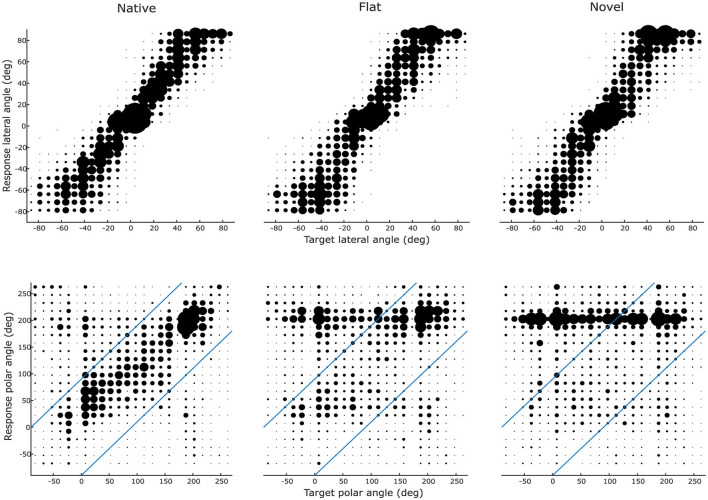
Directional localization patterns across cue types. Participants' (*N* = 14) angular responses for native (left column), flat (middle), and novel (right) HRTFs along the lateral **(top row)** and polar **(bottom)** dimensions. For visualization purposes, the lateral and polar angle ranges have been divided into 24 individual bins with a width of 15 degrees for polar angles and 7.5 degrees for lateral angles. Marker size indicates the frequency of responses in a certain bin. Responses from all participants (*N* = 14) were aggregated. Diagonal blue lines in the polar angle plots delineate the boundary between local (within boundaries) and global (beyond boundaries) polar error measures.

Figure 3 illustrates group-level lateral and polar localization response patterns for each cue-type in order to convey a first qualitative impression of the results. For the lateral angle, the scatter plots indicate high correspondence between target and response lateral angle (responses close to the main diagonal) for all three conditions. Larger differences across conditions are visible for the polar angle. In this dimension, high correspondence between target and response polar angle are only present for the native condition, whereas the flat and novel conditions led to a substantial degradation in relationship between target and response angles. For both flat and novel conditions, listeners exhibited a strong response bias toward the horizontal plane, primarily at their rear (around 180°).

**Figure 4:**
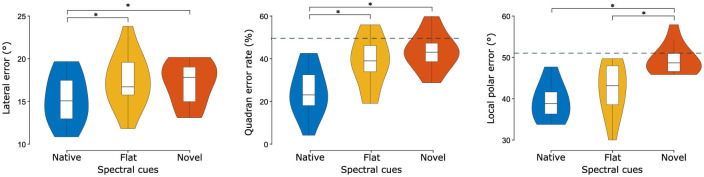
Localization performance metrics across cue types. Listeners (*N* = 14) performed substantially worse without their native spectral cues, especially along the polar dimension (local polar error and quadrant error rates). Central marks indicate the median and lower and upper hinges correspond to the first and third quartiles respectively. Lower and upper whiskers extend from the hinge to 1.5 times the IQR. Dashed gray horizontal lines indicate chance performance obtained by simulating a virtual experiment (Baumgartner et al., 2014). Note: chance performance for the lateral error is 52.56° but is not shown because it far exceeds the y-axis limit. Asterisks denote statistically significant differences (*p < 0.05).

For statistical evaluation, localization performance was quantified as lateral error, quadrant error rate, and local polar error (Figure 4). Spectral cue conditions significantly influenced the lateral error [*F*_(2, 26)_ = 12.89, *p* < 0.01, partial η^2^ = 0.50]). The effect of order (first half presentation vs. second half presentation) failed to reach significance [*F*_(1, 13)_ = 1.10, *p* = 0.31], as did the interaction between order and cue type [*F*_(2, 26)_ = 0.17, *p* = 0.83]. *Post-hoc* comparisons revealed that the mean lateral error measure was significantly lower in the native condition compared to both the flat [*d* = −2.07°, 95% CI (−3.26°, −0.88°), *p* < 0.01] and novel [*d* = −1.84°, 95% CI (−2.87°, −0.81°), *p* < 0.001] conditions. No significant difference was observed between flat and novel [*d* = 0.23°, 95% CI (−1.08°, 1.53°), *p* = 0.89] spectral cues.

Considering the quadrant error rate, we again observe a large effect of cue manipulation [*F*_(1.04, 13.56)_ = 20.07, *p* < 0.001, partial η^2^ = 0.61]. The effect of order again failed to reach significance [*F*_(0.52, 6.78)_ = 0.02, *p* = 0.90], as did the interaction between order and cue type [*F*_(1.05, 13.56)_ = 3.16, *p* = 0.08]. *Post-hoc* comparisons showed that performance in the native condition was significantly better than in both the flat condition [*d* = −14.25%, *p* < 0.01, 95% CI (−24.13%−4.38%)] and the novel condition [*d* = −18.99%, *p* < 0.001, 95% CI (−27.79%−10.19%)]. There was no significant difference in performance between flat and novel cue manipulations [*d* = −4.73°, 95% CI (−10.10°, 0.63°), *p* = 0.08].

Regarding the local polar error, cue manipulation again had a large effect [*F*_(2, 26)_ = 27.15, *p* < 0.001, partial η^2^ = 0.68]. There was no significant effect of order [*F*_(1, 13)_ = 0.44, *p* = 0.52], nor the interaction between order and cue type [*F*_(2, 26)_ = 0.70, *p* = 0.50]. *Post-hoc* comparisons found that the local polar error was significantly lower for native, compared to novel [*d* = −10.05°, 95% CI (−12.87°, −7.23°), *p* < 0.001] and also lower for flat, compared to novel [*d* = −6.96°, 95% CI (−10.56°, −3.35°), *p* < 0.001]. There was no significant difference between native and flat [*d* = −3.09°, 95% CI (−7.55°, 1.37°), *p* = 0.20] spectral cues.

We also investigated whether, and to what extent, performance was better (or worse) than chance-level. To that end, we simulated a virtual localization experiment with fully randomized responses (Baumgartner et al., 2014) and used this as a reference for normalization. The division of real and simulated error measures thus shows how similar the performance is to chance level, with 1 indicating chance performance and 0 indicating lack of any errors. When simulating the virtual localization experiment, an equal response distribution across all possible response locations inside the target range was assumed, while considering the actual target locations of the experiment. For the lateral error, all cue-types performed better than chance-level (native: 0.29; flat: 0.33; novel: 0.33). Regarding the local polar error, performance with native and flat spectral cues was, albeit a bit worse overall, again better than chance (native: 0.76; flat: 0.82). However, performance with novel cues was close to chance level (novel: 0.95). The quadrant error rate exhibits a similar pattern: evident better than chance performance for native and flat spectral cues (native: 0.49; flat: 0.77) and close to chance level with novel cues (novel: 0.87). Overall, polar-angle localization performance was poor and around chance level for novel spectral cues.

### 3.2 Externalization

**Figure 5:**
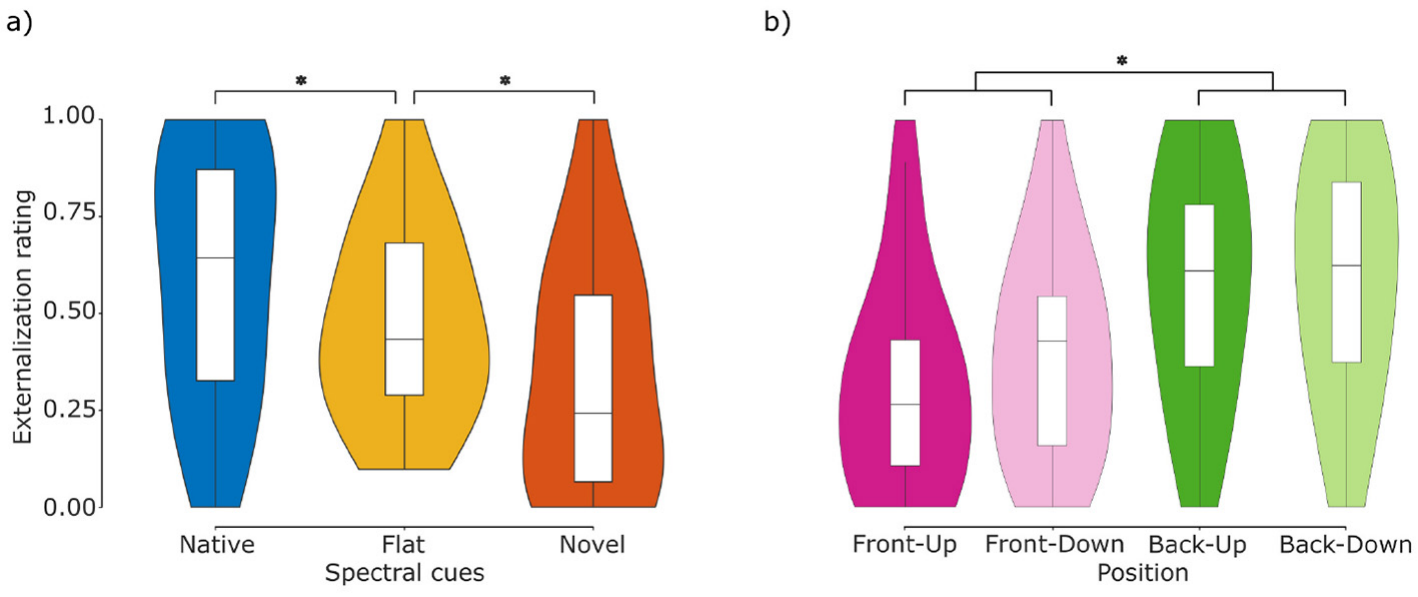
Externalization ratings. **(a)** Comparison of ratings across cue types. Listeners (*N* = 11) rated stimuli with flat spectral cues (middle column) as less externalized than with native spectral cues (left column), and more externalized than with novel spectral cues (right column). **(b)** Comparison of ratings across positions. Stimuli presented from the front (front-up and front-down) were rated as less externalized than stimuli presented from the rear (back-up and back-down). Central marks indicate the median and lower and upper hinges correspond to the first and third quartiles respectively. Lower and upper whiskers extend from the hinge to 1.5 times the IQR. Asterisks denote statistically significant differences (*p < 0.05).

We further analyzed the localizability of sounds under our different spectral cue manipulations and source positions in terms of the perceived degree of externalization. Figure 5a visualizes the distribution of the obtained externalization ratings across spectral cue types, indicating a gradual decay of externalization ratings with increasing deviation from the native reference. We analyzed those data by selecting the best mixed-effects model as outlined in Section 2.4.2. The model predicting externalization scores based on the two main effects of cue and position yielded the best fit (model #1 in Table 1). Compared to the intercept-only model, the BF approximation approach (Wagenmakers, 2007) revealed very strong evidence in favor of the cue-only model [*BF* = 27, 487.83; *Pr*(*H*_0_|*D*)>0.99] and strong evidence in favor of the position-only model [*BF* = 69.21; *Pr*(*H*_0_|*D*)>0.98], confirming a significant contribution of our main effects to explaining the data.

**Table 1:** Externalization model summary.

**Models**			**dBIC**		
**1**. *Ext*~(*cue*+*pos*)|*sbj*			0		
**2**. *Ext*~(cue*pos)|*sbj*			14.1		
**3**. *Ext*~(*cue*)|*sbj*			15.1		
**4**. *Ext*~(*pos*)|*sbj*			27.1		
**5**. *Ext*~1|*sbj*			35.1		
**Fixed effects coefficients (model 1)**	**Est**	**SE**	*z*	**Pr(**>|*z*|**)**	
(Intercept) reference cue = nat, pos=FU	0.06	0.23	0.26	0.80	
cue = flat	–0.88	0.24	–3.76	< 0.01	***
cue = nov	–1.83	0.25	–7.43	< 0.01	***
pos = FD	0.28	0.27	1.05	0.30	
pos = BU	1.34	0.27	4.88	< 0.01	***
pos = BD	1.33	0.27	4.85	< 0.01	***
**Predictions (model 1) for Ext**	**Pos:**	**FU**	**FD**	**BU**	**BD**
cue = nat		0.51	0.58	0.80	0.80
cue = flat		0.30	0.37	0.63	0.62
cue = nov		0.15	0.18	0.39	0.39

Several models were estimated and compared relative to the winning model #1 in terms of BIC difference. Smaller BIC values indicate better model fit. For the selected model #1, fixed effects are reported with model estimates (Est), standard errors (SE), *z*-ratio, corresponding *p*-values and model predictions. Fixed effects are relative to the respective reference levels (native for cue, FU for position). Ext = externalization rating (0–1); cue = spectral cue type (native, flat, novel); pos = position (FU, FD, BU, BD); sbj = participant ID (1–11). Asterisks denote statistically significant differences (^***^p < 0.001).

Regarding the main effect of spectral cue type, the estimated marginal means suggest a gradual decay of externalization perception from native, over flat to novel spectral cues. That is, native stimuli were rated as the most externalized [native = 0.67, 95-CI(0.61, 0.74)], followed by flat stimuli [flat = 0.48, 95-CI(0.41, 0.55)] and finally novel stimuli [novel = 0.28, 95-CI(0.22, 0.34)]. *Post-hoc* pairwise comparisons revealed that the differences between all three levels of cue types were significant (native—flat: *z* = 3.84, *p* < 0.01; native—novel: *z* = 8.26, *p* < 0.01; flat—novel: *z* = 4.10, *p* < 0.01). These differences in externalization perception between different spectral cue types are in-line with the model-predicted ratings (Figure 1c) and thus support the expected motion perception in the looming bias paradigm. This enables to test the Spatial Perception Hypothesis against the Acoustic Hypothesis.

Results regarding the main effect of position are shown in Figure 5b. The estimated marginal means indicated that stimuli presented from the frontal hemifield were rated as less externalized [FU = 0.32, 95-CI(0.25, 0.40); FD = 0.38, 95-CI(0.30, 0.46)] than stimuli presented from the rear hemifield [BU = 0.61, 95-CI(0.53, 0.69); BD = 0.61, 95-CI(0.53, 0.68)]. *Post-hoc* pairwise comparisons showed no significant differences between the positions within frontal and rear hemifields (FU–FD: *z* = –1.05, *p* = 0.72; BU–BD: *z* = 0.04, *p*>0.99), but significant differences between them (FU–BU: *z* = –5.13, *p* < 0.01; FU–BD: *z* = –5.09, *p* < 0.01; FD–BU: *z* = –4.02, *p* < 0.01; FD–BD: *z* = –3.98, *p* < 0.01).

### 3.3 Looming bias

**Figure 6:**
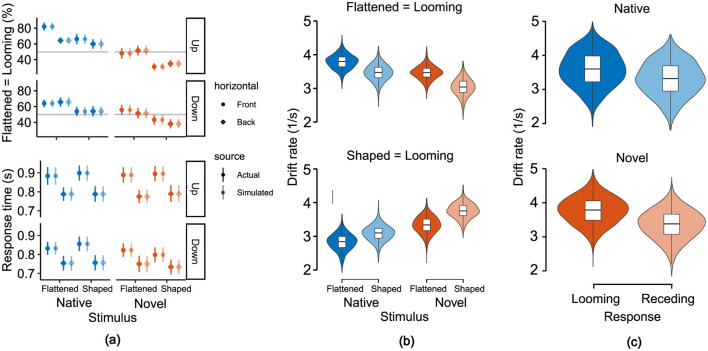
Model-based examination of behavioral responses shows increased speed of evidence accumulation for looming responses. **(a)** Comparison of response times and proportion of flattened-to-looming-match responses between actual data and simulations of the fitted linear ballistic accumulator model. Symbols represent means and error bars indicate standard errors over participants. **(b)** Stimulus-based sorting of posterior distributions of drift rate estimates reveals the speed of evidence accumulation for motion direction, cue type and stimulus-to-response-match (flattened matched with looming and shaped with receding in top panel, vice versa for bottom panel). **(c)** Response-based sorting of posterior distributions of drift rate estimates reveals faster speed of evidence accumulation for looming responses, regardless of stimulus-to-response-match and cue type.

Figure 6a shows the mean response times and the proportion of flattened-to-looming-match responses. In the native flattened condition, participants indicated “looming” in most cases (60%–80%) and in the native shaped condition, they primarily responded “receding” (50%–60%). In the novel flattened condition, however, participants responded mostly random (50% “looming” and 50% “receding”), whereas in the novel shaped condition they mostly responded with “looming” (60%–70%), indicating a perceptual switch between native and novel spectral cue conditions.

**Figure 7:**
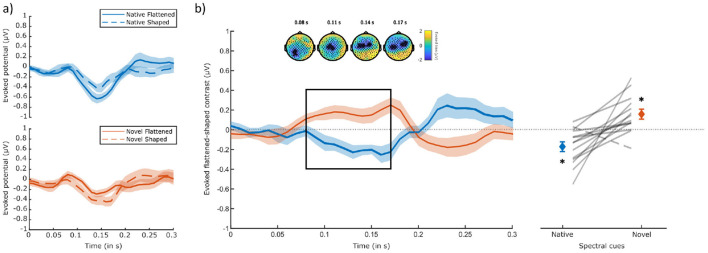
ERPs evoked by spectral shape transitions. **(a)** Scalp potentials evoked by flattened (solid lines) and shaped (dashed lines) sounds for both native (top) and novel (bottom) spectral cues, averaged over the channels of interest (all 38 channels that contributed to the cluster). Shaded areas indicate the standard error of the mean. **(b)** Left panel: evoked flattened-shaped contrast for both native and novel spectral cues, averaged over the cluster's channels. Significant time window of difference (native – novel) is highlighted with a black rectangle and occurred around typical N1 time range. Shaded areas indicate the standard error of the mean. Above, topological plots of the significant cluster at four time points. Contributing channels are highlighted (*). Right panel: amplitudes averaged per cluster on the group (bars) and participant (horizontal lines) levels. All listeners (*N* = 14), except one, exhibited larger negative amplitudes for flattened sounds, comparing to shaped sounds with native spectral cues and larger negative amplitudes for shaped sounds, compared to flattened sounds with novel spectral cues. Error bars denote standard errors of the means.

Before constructing the neurobehavioral model, results of the ALB paradigm were first investigated based on EEG data only. Cluster-based permutation tests showed no significant differences between flattened and shaped stimuli within cue-type (*p* = 0.072 for a negative cluster in the native condition ranging from 100 to 180 ms after correction for two-tailed testing). Comparing the differences between flattened and shaped stimuli between cue types, however, revealed a significant amplitude difference between the native and novel flattened-shaped contrasts in the N1-related range of 80–170 ms (clusterstat = –294.0344, *p* = 0.04) after motion onset. The native condition was characterized by a more negative N1-related-cluster amplitude for flattened, compared to shaped stimuli. In contrast, the novel condition was instead characterized by a more negative amplitude for shaped, compared to flattened stimuli (Figure 7a), in line with the externalization-based ALB elicitation hypothesis (Figure 1c) and participant's externalization ratings for the different spectral cue manipulations (Figure 5). Topographically, this difference across cue types emerged over left occipital electrodes and subsequently propagated toward central electrodes. At around 140 ms, the cluster became spatially bimodal and fronto-temporally lateralized (Figure 7b, right-most topographies). We further confirmed that both the native and novel evoked differences between flattened and shaped stimuli within the N1-related cluster of difference were significantly different from zero (native: *t* = −3.46, *p* < 0.01; novel: *t* = 3.16, *p* < 0.02, see Figure 7b, right panel). A two-way repeated measures ANOVA further revealed a significant main effect of spectral cue on the cluster amplitude of the evoked native-novel difference [*F*_(1, 13)_ = 16.4, *p* < 0.001, partial η^2^ = 0.56]. There was no significant effect of position [*F*_(3, 39)_ = 1.52, *p* = 0.22], nor was there a significant interaction between spectral cue and position [*F*_(3, 39)_ = 0.46, *p* = 0.71].

We then investigated the behavioral results of the motion discrimination task by comparing different LBA models used to describe the speed of evidence accumulation in terms of drift rates. We started with identifying the best purely behavioral LBA model and then tested the benefit of adding the trial-by-trial cluster amplitudes (see Section 3.3) as an additional neural regressor:


(2) $$\begin{array}{l} v\sim match*direction*cue+vertical*horizontal+eeg \end{array}$$

Our comparison identified this full model where the drift rate was modulated by all five acoustic and behavioral factors (i.e., flattened-stimulus-to-looming-response match, cue type, motion direction, vertical and horizontal positions) and the additional neural marker as the best fit. The quality of fit was lower without the neural marker (dBPIC = 2) and the preceding selection of the purely behavioral reference model showed that it clearly outperformed simpler alternatives (intercept only: dBPIC = 2,999; match only: dBPIC = 2,701; match and direction: dBPIC = 2,318; match, direction and cue: dBPIC = 1,129) as well as an alternative regressand (response threshold instead of drift rate: dBPIC = 99). The benefit of adding the neural marker was also verified against surrogate data matching the mean and SD of the cluster scores (dBPIC = 12). In Figure 6a, we report a direct comparison between the real and simulated behavioral data obtained from the winning model.

**Figure 8:**
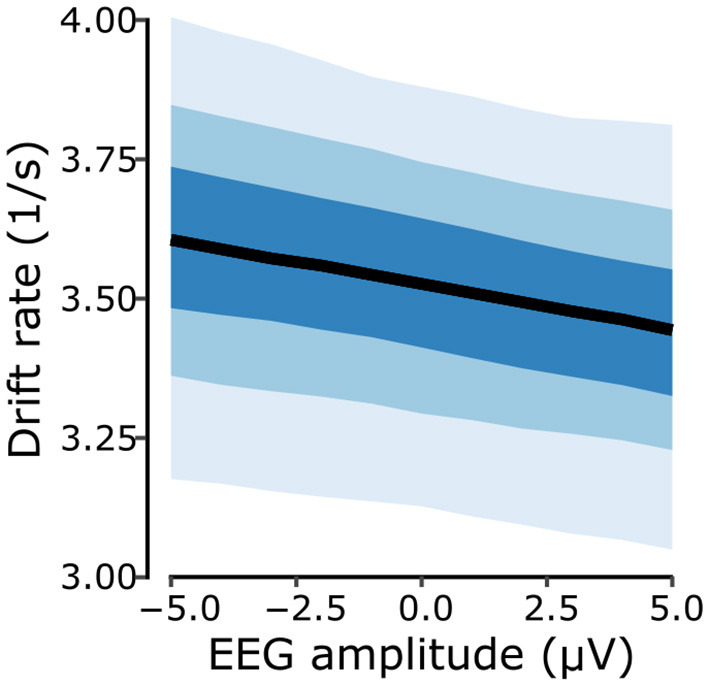
Drift rate as a function of the N1-related EEG cluster amplitude. A higher drift rate, i.e. a faster evidence accumulation process is associated with a more negative N1-related cluster amplitude. Shaded areas around the fitted drift rate values indicate the 50%, 80%, and 95% credible intervals (50% CI = darkest blue).

We then used the model predictions to assess the strength of the ALB in terms of drift rates. The results demonstrate a dependence on perceived motion direction (Figures 6b, c). For correct responses, drift rates resulted higher for flattened compared to shaped stimuli [*P*_Δ>0_ = 0.995, with Δ = 0.362*s*^−1^, 95%-CI (0.093, 0.658)]. For incorrect responses, drift rates resulted smaller for flattened compared to shaped stimuli [*P*_Δ < 0_ = 0.970, with Δ = −0.339*s*^−1^, 95%-CI (–0.648, 0.017)], indicating that looming responses consistently exhibited higher drift rates compared to receding responses. Furthermore, the analysis revealed that cue type modulates drift rates differently based on response match. For correct responses, the native condition yielded a greater drift rate compared to the novel condition [*P*_Δ>0_ = 0.951, with Δ = 0.356*s*^−1^, 95%-CI (–0.079, 0.793)]. Conversely, for incorrect responses, drift rates for the native condition reported smaller values than those from the novel condition [*P*_Δ < 0_ = 0.991, with Δ = −0.590^−1^, 95%-CI (–1.088, –0.106)]. Finally, we inspected the magnitude of the fitted linear coefficient associated with the magnitude of the N1-related EEG cluster for the prediction of the model's drift rate which corresponded to –0.017 1/μ*Vs* with a 95%-CI (–0.039, 0.005), and with a 0.942 probability of it being a negative value. Figure 8 illustrates the linear relationship between the EEG predictor (N1-related EEG cluster amplitude) and the fitted drift rates, revealing that faster evidence accumulation is associated with a more negative EEG amplitude. This result, alongside the model comparison, further supports the notion that there is a coherent relationship between the neurophysiological marker and the behavioral outcome.

## 4 Discussion

The present study set out to clarify whether the biases observed in previous looming studies are truly a spatial phenomenon. Do sounds need to be localizable in order to elicit the (spectral) ALB or are mere stimulus characteristics sufficient? To answer this question, we first proposed an HRTF manipulation method to obtain novel spectral cues that provided the same amount of spectral shaping as native cues while significantly deteriorating sound localizability in terms of directional localization and externalization perception. Then, we demonstrated that biases in early perceptual processing (N1-related cluster amplitudes) evoked by novel spectral cues are consistent with the direction of externalization change rather than stimulus shaping. Finally, we confirmed that the behavioral speed of evidence accumulation is generally higher for looming responses and showed that this behavioral effect can better be explained by integrating the EEG marker.

### 4.1 Novel spectral cues degrade directional localization performance and perceptual externalization

We demonstrated that localization performance with novel spectral cues was significantly impaired compared to performance with native cues across all error measures (lateral error; local polar error; quadrant error rate). This indicates that established spatial associations between stimulus characteristics and locations were successfully broken apart. Furthermore, we also observed diminished local polar localization performance for novel cues compared to flat cues, which is also reflected in the fact that performance with novel cues was close to chance performance, whereas performance with flat cues was still better than chance.

This raises the question of why participants were still able to localize sounds in the polar dimension reliably at least to some degree with flat spectral cues. A possible explanation is that our HRTF manipulation only targeted frequencies above 1 kHz (see Section 2.3), thus low frequency information provided by torso reflections was still available to participants and possibly provided some usable elevation (Algazi et al., 2001), externalization (Zotter and Frank, 2018) and front-back discriminatory cues (Asano et al., 1990). The additional presence of contradictory, i.e., inverted, high frequency spectral cues in the novel cue condition could have potentially interfered with the information provided below 1 kHz, thus, leading to worse performance and a stronger degradation of externalization than in the flat condition, where low frequency cues were the only available ones. We also observed a response bias toward the horizontal plane with flat and novel spectral cues, primarily at participant's rear (around 180°). A response bias such as this is, also referred to as low elevation gain (slope of the stimulus-response relation) (Zwiers et al., 2003) under perceptual uncertainty, i.e. with impoverished spectral cues, is in line with previous work reporting similar patterns (Wanrooij and Opstal, 2005; Zonooz et al., 2018, 2019). A second possible explanation could arise from an implementation issue in the experimental design. Due to a miscommunication, participants were tested using a fixed order of spectral cue conditions—native, flat, and novel—which coincides with the observed performance ranking. However, since this fixed order was repeated three times, it also included native after novel blocks and thus is rather unlikely to account for the pronounced differences between conditions.

Moreover, lateral localization performance and quadrant error rate was also significantly reduced with flat spectral cues, compared to native spectral cues. Even though the impairment was rather small, reduced lateral localization abilities with flat spectral cues is in-line with a more recent, updated, view on the contribution of spectral cues for the lateral dimension. Traditionally, lateral sound localization was thought to almost exclusively rely on binaural cues, such as interaural time- and level differences, with monaural spectral cues thought to only contribute very little (Macpherson and Middlebrooks, 2002). However, recent work (Stevenson-Hoare et al., 2022) has challenged this idea by demonstrating an apparent front/back asymmetry in sound localization precision measures, which is especially strong for oblique azimuths. That is, the pinna introduces a high-frequency spectral cue that enhances lateral sound localization in the frontal hemifield only. Hence, contemporary computational models on human sound localization include both monaural and binaural cues as joint contributors to lateral and polar localization performance predictions (Barumerli et al., 2023). By flattening individualized HRTFs, these additional cues were no longer available to our participants, potentially explaining their diminished lateral localization performance with flat HRTFs compared to native HRTFs.

Furthermore, we also demonstrated that the spectral manipulation of individualized HRTFs degraded the perceived externalization of sound sources. An externalization breakdown was observed for both flat and novel HRTFs. Stimuli based on novel HRTFs were rated as even less externalized than stimuli based on flat ones, indicating a shift toward an internalized percept with novel, spectrally inverted HRTFs and a gradual decay of externalization. This decay is consistent with a template-based model of externalization under static listening conditions (Baumgartner and Majdak, 2021). Under this framework, perceptual externalization depends on comparing observed auditory cues and listener-specific templates in memory. When incoming sounds deviate from the established reference template, externalization perception is predicted to gradually decrease with increased deviation magnitude. In the present study this assumption would thus also predict a significant, but smaller, difference between native (e.g., template-matching) and flat (e.g., template-deviation) stimuli, compared to the difference between native and novel stimuli consistent with our observation.

### 4.2 ALB elicitation conforms with spatial percept

Based on the present results we conclude that ALB elicitation was consistent with the listeners' perceived changes in externalization rather than triggered by inherent acoustical features independent of auditory space. In line with the template-based model predictions (Baumgartner and Majdak, 2021), participants reported less externalization for novel stimuli compared to flat and native stimuli. Furthermore, they tended to exhibit a low rate of shaped-stimulus-to-receding-response match, indicating that their perception of novel stimuli seems to have been reversed compared to native stimuli. Stimuli that exhibited a reduction of perceptual externalization during the short stimulus transition were perceived as looming (e.g. native flattened and novel shaped), and vice versa, stimuli that contained an increase of perceptual externalization were perceived as receding (e.g. native shaped and novel flattened). The speed of evidence accumulation, i.e. the drift rate, was also consistently higher for looming responses, regardless of stimulus-to-response-match. Taken together, our data support the hypothesis that ALB elicitation relies on spatial perceptual associations, rather than purely acoustic properties (see Figures 1b, c).

Although the current study demonstrates that ALB elicitation conformed with participant's spatial perception, due to its correlational nature we cannot clearly pinpoint the causal dependencies between bias elicitation, sound localizability and externalization perception in particular. A follow-up learning study investigating the generalizability from sound localization to ALB elicitation with novel spectral shape cues (Greif et al., 2024) will address this.

### 4.3 Early perceptual processing biases affect speed of evidence accumulation

Neural markers of ALB were identified between 80 ms and 170 ms after motion onset via cluster-based permutation testing and confirmed to be behaviorally relevant via LBA modeling. The latency and topography of that marker closely relates to the N1 ERP component and is consistent with previously reported results on ALB and motion perception (Ignatiadis et al., 2021, 2024a). The N1 component is an established marker of early perceptual processing, implicated to play a central role in attention allocation via sensory gain control (Getzmann, 2011; Getzmann and Lewald, 2010; Hillyard et al., 1998). That is, attended stimuli tend to exhibit an increased N1 amplitude compared to unattended stimuli. In the context of our study, sounds exhibiting a decay of externalization were predominantly perceived as looming, captured attention, and thus facilitated ALB elicitation. Interestingly, previous work has demonstrated this neural bias toward looming sounds also while attention was diverted, in a passive listening mode (Ignatiadis et al., 2024a), highlighting the high salience and attentional capture potential of approaching sound sources.

Incorporating the trial-by-trial N1-related EEG cluster amplitudes in the LBA model improved its explanatory power. Yet, the overall benefit was rather small (dBIC = 2), suggesting that this single-trial maker was quite noisy and supposedly incomplete. Alternative neurophysiological markers such as ERP latency, oscillatory power, or a joint metric, should thus be considered. Previous corticocortical connectivity analyses have identified bottom-up projections between temporal, parietal and the frontal regions as a possible neural network involved in the elicitation of the ALB (Ignatiadis et al., 2021). Specifically, connectivity among regions along the ventral stream, traditionally coined the “what” stream in visual and auditory processing (Saur et al., 2008), seems to play an essential role in ALB elicitation (Ignatiadis et al., 2024b). This makes it a suitable target for future work incorporating more sophisticated neural ALB markers (e.g. phase locking value). Implementing more flexible joint modeling methods with non-linear relationship assumptions (Palestro et al., 2018; Nunez et al., 2024) could also further improve the present model. Translating the present work into more immersive environments, e.g. via VR-based applications using more realistic sounds, could also lead to a stronger perceptual impression of spectral looms and recedes, potentially increasing the observed effects (Deng et al., 2019; Getzmann and Lewald, 2010) and strengthening the relationship between behavioral measurement and neural marker.

### 4.4 Limitations

A key limitation of the present study is the relatively small sample size, which may reduce the statistical power to detect small or moderate effects. This limitation implies that non-significant findings, such as the lack of EEG differences between flattened and shaped stimuli within cue-type, should be interpreted with caution, as they may reflect insufficient power rather than a true absence of effect. Nevertheless, the study provides initial evidence that the emergence of auditory looming bias requires spatial perceptual associations to be established.

Another limitation concerns the low ecological validity of stimuli. As in previous studies (Baumgartner et al., 2017; Ignatiadis et al., 2024a), we here isolated spectral from intensity cues to hone in on the contribution of spatial perception to ALB, but it is worth noting that this isolation of cues does not happen outside of the laboratory and is expected to limit the extent of ALB (Bach et al., 2009) and attention control (Deng et al., 2019). Secondly, the spectral cue manipulations to elicit looming and receding sensations happened on a very rapid time scale. Such a rather instantaneous change in perceived distance has the advantage of providing a clearly defined event of interest for the analysis of EEG data, but it may limit the generalization of our results to the real world where changes in distance usually occur more gradually. Finally, our experimental setting did not allow participants to move during stimulus presentation because this would render the stimuli even more implausible. Listeners naturally engage in self-motion to resolve perceptual ambiguities (Wallach, 1940), consistent with principles of active inference (Friston et al., 2016). Future work is needed to develop more naturalistic experimental settings with high stimulus control for neuroscientific investigations.

### 4.5 Outlook

The here presented results establish a strong link between spectrospatial associations and looming/receding perception, implying that any disruption of natural spectral cues would also disrupt motion perception in general, leading to potentially dangerous situations for anyone wearing hearing aid or hearing protection devices. Furthermore, the preservation of natural cues, or at least providing a sufficiently good approximation, seems integral for developing immersive virtual realities that provide realistic auditory experiences. Actively manipulating spectral cues provided to listeners could also provide unique and interesting opportunities for the development of gamified environments and/or audio design.

## Data Availability

The original contributions presented in the study are publicly available. This data can be found here: https://osf.io/f5wx2/.
